# Case Report: Acute pancreatitis obscured by paralytic ileus in a patient with extensive burns

**DOI:** 10.3389/fsurg.2026.1715967

**Published:** 2026-04-30

**Authors:** Hui Wang, Da Wang, Jun Liu

**Affiliations:** 1 First Ward of Department of Burn, Gansu Provincial Hospital, Lanzhou, China; 2 Burns Clinical Medical Center, Gansu Provincial Hospital, Lanzhou, China

**Keywords:** acute pancreatitis, burns, case report, critical patients, paralytic ileus

## Abstract

Extensive burns are associated with numerous severe complications. While paralytic ileus is relatively common, the co-occurrence of acute pancreatitis (AP) is rare, clinically insidious, and highly fatal. Diagnosis is particularly challenging due to masking by critical illness and coexisting ileus. We present a 32-year-old male with 65% TBSA flame burns and inhalation injury, admitted in hypovolemic shock. Resuscitation followed Parkland protocol, and escharotomy was performed. On day 2, abdominal distension and reduced bowel sounds suggested paralytic ileus, managed with nasogastric decompression and parenteral nutrition. By day 5, epigastric pain developed. Serum amylase and lipase (peaking at 580.38 U/L) and urinary amylase were elevated. Abdominal CT and MRI confirmed AP. Treatment included strict nil-by-mouth, aggressive support, and somatostatin. Serum lipase normalized with clinical improvement, while urinary amylase remained elevated until day 13. The patient recovered fully after skin grafts and was discharged. This case underscores that AP can be a lethal burn complication often obscured by ileus. Serum lipase is more reliable than urinary amylase for diagnosis and monitoring, especially with concurrent kidney injury. Vigilance, timely imaging, and multimodal support are critical. Persistent urinary amylase may reflect renal dysfunction rather than ongoing pancreatitis.

## Introduction

Extensive burns represent a devastating trauma, and their management remains a major challenge in critical care medicine. Despite significant advances in fluid resuscitation strategies and intensive care techniques, patients still face high mortality risks due to complex pathophysiological changes, primarily attributable to severe complications such as burn shock, sepsis, and multiple organ dysfunction (1). Among these complications, gastrointestinal dysfunction is particularly prevalent (1), with studies indicating an incidence rate as high as 45.4% in extensive burn patients, most commonly presenting as paralytic ileus (1). However, acute pancreatitis (AP)—a complication characterised by insidious onset, rapid progression, and extremely high mortality—remains relatively uncommon following extensive burns (2). The clinical manifestations of the condition are easily masked by the patient's critical systemic condition (e.g., sedation, wound pain, concomitant intestinal obstruction), leading to missed or delayed diagnosis and missed intervention opportunities. In view of the considerable diagnostic challenges and the grave prognosis of AP following extensive burns, heightened clinical vigilance is imperative. This report meticulously documents the effective management of acute pancreatitis and intestinal obstruction subsequent to a burn injury encompassing 65% TBSA. The present study aims to explore the potential underlying mechanisms and provide guidance for early clinical recognition and intervention of such critical complications. To this end, a thorough description of the clinical progression, diagnostic approach, and treatment strategy is provided, combined with a literature review.

## Case presentation

A 32-year-old male patient with extensive burns, swelling and pain across multiple body areas was admitted to the Burn Unit at Gansu Provincial People's Hospital. The patient sustained these injuries following an electrical panel fire that occurred seven hours prior to admission. The patient presented with severe burns covering a substantial proportion of the body surface area, including flame burns to the head, face, neck, trunk, and extremities, amounting to approximately 65% of the total body surface area (TBSA). The injuries sustained comprised 15% second-degree burns and 50% third-degree burns, accompanied by respiratory tract burns. The patient exhibited no antecedent health concerns or history of long-term medication use. The patient had been without sustenance or hydration since the injury occurred and was transferred to our hospital approximately seven hours later. Upon admission, the patient exhibited the following vital signs: a heart rate of 134 beats per minute, a blood pressure of 90/55 mmHg, a respiratory rate of 31 breaths per minute, and an oxygen saturation level of 98%. The presence of dark brown urine was observed. The laboratory tests revealed the following results for the patient: a haemoglobin level of 214 g/L; a platelet count of 593 × 10^9^ /L; and electrolyte levels. The concentrations of potassium (K^+^), sodium (Na^+^), chloride (Cl⁻), calcium (Ca^+^) and magnesium (Mg^+^) were determined to be 4.12, 147.62, 116.37, 1.66 and 0.71 mmol/L, respectively. The urinalysis revealed that the colour of the sample was brown, its clarity was cloudy, and the protein level was 2+ (normal range: 0–1+). The bilirubin level was 1+ (normal range: 0–1+), the specific gravity was 1.020, and the presence of occult blood was 3+ (normal range: 0–1+). The presence of ketones was also noted. +; Nitrites: +. Following admission, the Parkland formula of crystalloid and colloid fluid resuscitation for shock management was continued. Prophylactic antimicrobial therapy was initiated with the administration of Cefoperazone/Sultamicillin 3 g once every 12 h. The therapeutic approach encompassed the administration of Omeprazole 40 mg once daily to address the underlying acidity. Emergency debridement and eschar removal of burn wounds was performed a total of three hours after admission. Intraoperative fluid infusion: 3,000 mL; plasma infusion: 1,000 mL. Postoperative continuation of relevant supportive therapy.

**Figure 1:**
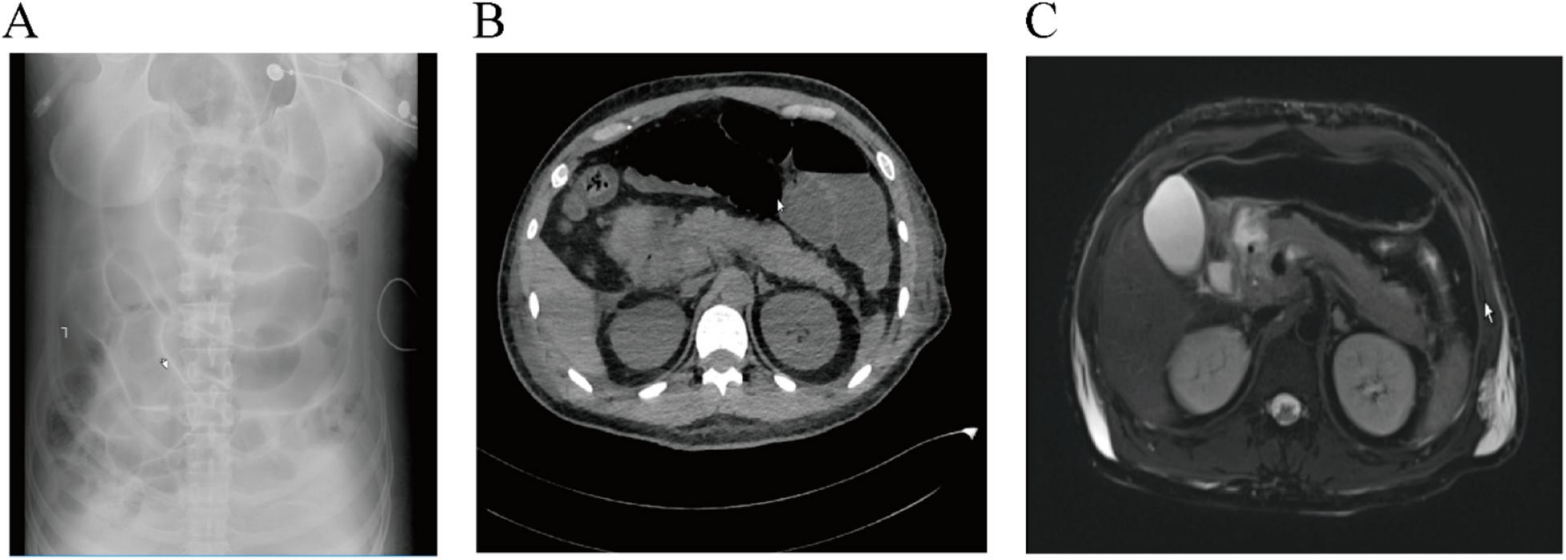
**(A)** bedside abdominal x-ray reveals small bowel dilatation with gas accumulation and multiple dilated bowel loops; **(B)** abdominal CT scan shows pancreatic enlargement with blurred surrounding fat planes; **(C)** abdominal MRI scan demonstrates a full-appearing pancreas with surrounding cotton-wool-like long T2 signals and diffuse restricted diffusion in the parenchyma.

On the second day after admission, the patient exhibited symptoms of abdominal distension and decreased bowel sounds, with bowel sounds measuring between 1 and 2 times per minute. An urgent bedside abdominal x-ray revealed small bowel dilatation with gas accumulation, multiple dilated bowel loops, and signs of intestinal obstruction (Figure 1A). The diagnosis of intestinal obstruction was confirmed, and a decision was made to initiate symptomatic supportive treatment, including gastrointestinal decompression, parenteral nutrition support, and gastric acid suppression. The patient's abdominal distension and pain gradually resolved.

However, on the fifth day after admission, the patient complained of upper abdominal pain. An urgent blood test revealed markedly elevated serum lipase levels, along with elevated serum lipase and urinary amylase (Table 1). Concurrent urgent abdominal CT revealed the following: an enlarged pancreatic volume with blurred peripancreatic fat margins; post-upper GI tube placement; marked abdominal gas, fluid accumulation, and intestinal dilatation; prominent annular thickening of the lower rectal wall. The findings indicated the presence of pancreatitis and intestinal obstruction (Figure 1B). Subsequent MR imaging revealed a distended pancreatic contour accompanied by surrounding T2 hyperintensity resembling cotton wool, along with diffuse parenchymal restriction, thereby confirming the diagnosis of acute pancreatitis (Figure 1C). Following the diagnosis, the patient underwent a course of symptomatic supportive management, which included fasting, gastrointestinal decompression, parenteral nutrition, and acid suppression. The patient was administered a continuous somatostatin infusion at a rate of 250 μg/h. There was a gradual improvement in symptoms after a period of two weeks. Serum lipase levels exhibited a downward trend, reaching a nadir within a fortnight and subsequently stabilising. Despite the manifestation of clinical improvement, urinary amylase levels remained persistently elevated until day 13 after admission, at which point they began to gradually decline, thus demonstrating asynchrony with the clinical course (see Table 1 for details). The abdominal pain resolved by day 17, with bowel sounds at 4–5 per minute, thus allowing gradual resumption of enteral nutrition. Following a series of skin grafting procedures, the patient exhibited a positive response, with the majority of wounds demonstrating signs of healing. Consequently, the patient was discharged from the hospital.

**Table 1:** Laboratory test indicators after admission.

Time after admission	Day 5	Day 7	Day 10	Day 13	Day 16	Day 19
Urine amylase (U/L)	396.55	1163.93	1652.3	1948.82	1637.65	759.52
Serum lipase (U/L)	580.38	520.82	450.54	290.73	150.01	75.67
Hemodiastase (U/L)	118.82	133.86	167.86	174.86	193.25	94.07

## Discussion

This study reports a case of paralytic ileus and acute pancreatitis (AP) complicating extensive burns (65% total body surface area). Although rare, this complication manifests with critical illness, insidious clinical progression, and significant diagnostic and therapeutic challenges (2). The clinical course, characteristic laboratory parameter dynamics, and treatment outcomes of this case provide important evidence for further exploration of the pathogenesis, diagnostic criteria, and management strategies of post-burn AP.

At the time of admission, the patient exhibited the symptoms of classic hypovolemic shock, which is characterised by hemoconcentration with elevated hemoglobin (214 g/L), hypotension, myoglobinuria, and acute kidney injury (serum creatinine 262.24 μmol/L). This hemodynamic disturbance formed the key pathophysiological basis for subsequent complications. The pancreas, an organ characterised by its low perfusion, exhibits heightened sensitivity to ischemia. Insufficient visceral perfusion caused by severe shock can directly impair pancreatic microcirculation, leading to acinar cell damage and intracellular proenzyme activation, thereby initiating the pathological process of AP (1, 3, 4). Concomitant aggressive fluid resuscitation and emergency surgical debridement, while successful in saving the patient's life, may also result in the induction of ischemia-reperfusion injury (IRI). During reperfusion, substantial oxygen radical generation further exacerbates cellular injury and activates systemic inflammatory responses, constituting a “second hit” to the pancreas. In this case, AP-related symptoms and elevated enzymatic markers emerged on the fifth day post-admission, aligning with the timeline following resuscitation and surgery, thereby supporting the involvement of the aforementioned mechanisms in disease development.

This case vividly illustrates the diagnostic complexity of acute pancreatitis following burns. The early symptoms and signs of the condition are easily masked by concurrent paralytic ileus. Of particular significance was the observation that the dynamic fluctuations in pancreatic enzyme levels exhibited a discernible pattern: namely, fluctuations in serum lipase levels (peaking at 580.38 U/L) exhibited a close correlation with both clinical deterioration and improvement, thereby reinforcing its reliability as a diagnostic marker for AP and an indicator of disease activity. This case vividly illustrates the diagnostic complexity of acute pancreatitis following burns. The early symptoms and signs of the condition are easily masked by concurrent paralytic ileus. Of particular significance was the observation that the dynamic fluctuations in pancreatic enzyme levels exhibited a discernible pattern: namely, fluctuations in serum lipase levels (peaking at 580.38 U/L) exhibited a close correlation with both clinical deterioration and improvement, thereby reinforcing its reliability as a diagnostic marker for AP and an indicator of disease activity. However, urinary amylase exhibited a distinctly divergent trajectory: its peak appeared delayed and declined slowly, not beginning to decrease until the 13th day of hospitalization. This critical finding cannot be explained by persistent pancreatic inflammation but is more likely associated with acute tubular injury caused by shock and rhabdomyolysis (resulting from extensive muscle burns). Impaired renal function had a substantial impact on amylase clearance, resulting in prolonged retention of the enzyme in both blood and urine (5). Consequently, in patients with burn injuries who also exhibit acute kidney injury, reliance on urinary amylase as a diagnostic or monitoring marker may result in misinterpretation. Serum lipase levels and imaging findings should be accorded priority.

The utilisation of imaging studies proved instrumental in arriving at a definitive diagnosis in this particular instance. Abdominal CT and MRI revealed pancreatic enlargement, blurred peripancreatic fat margins, and diffuse parenchymal consolidation—findings consistent with the imaging criteria for acute pancreatitis. From a pathophysiological perspective, the systemic inflammatory response syndrome (SIRS) triggered by severe burns has the potential to result in simultaneous impairment to gastrointestinal and pancreatic function. It is hypothesised that compromised intestinal barrier function may result in bacterial/endotoxin translocation, which, in turn, activates hepatic Kupffer cells via the portal venous system. This has been shown to trigger a massive release of inflammatory mediators that remotely affect the pancreas^2^, forming a vicious “gut-pancreas axis” cycle that accelerates AP progression (6–8).

The therapeutic success of this case was achieved through comprehensive supportive treatment facilitated by multidisciplinary collaboration, adhering to core principles including: Firstly, it is essential to ensure complete pancreatic rest, which can be achieved through strict fasting and effective gastrointestinal decompression. Secondly, precision fluid management is crucial for balancing burn resuscitation and stabilising the internal environment. Thirdly, early parenteral nutrition support is necessary to ensure adequate nutrition. Fourthly, the rational use of somatostatin to inhibit pancreatic exocrine function is important to manage the condition. Finally, aggressive management of primary burn wounds is essential to control infection sources. This finding indicates that in the absence of specific therapeutic agents, early recognition, organ function support, and infection control can create favourable conditions for pancreatic self-repair.

This study is a single case report with inherent limitations, precluding the establishment of a definitive causal relationship between burns and AP. The proposed mechanisms also contain speculative elements. Nevertheless, the case's value lies in clearly illustrating the clinical characteristics and diagnostic challenges of post-burn AP, particularly emphasising key points in interpreting enzyme dynamics monitoring and the importance of multimodal management, providing valuable insights for clinicians.

## Conclusion

Acute pancreatitis subsequent to extensive burns is an insidious and potentially life-threatening complication. Clinicians must remain highly vigilant, particularly when patients present with abdominal pain that cannot be explained solely by intestinal obstruction or when their condition deteriorates. An aggressive diagnostic strategy is advocated for such patients, with serum lipase monitoring given priority over urinary amylase, and the performance of abdominal CT scans given priority. Early recognition, comprehensive understanding of the underlying pathophysiological mechanisms (including hypoperfusion, ischemia-reperfusion injury, and the impact of rhabdomyolysis on renal function), and the implementation of bundled comprehensive supportive care are pivotal to improving outcomes. In order to achieve a more complete understanding of its epidemiological characteristics and precise pathophysiological mechanisms, further larger-scale clinical studies are required.

## Data Availability

The original contributions presented in the study are included in the article/Supplementary Material, further inquiries can be directed to the corresponding author.
